# Cognitive Reserve in Amyotrophic Lateral Sclerosis: A 2‐[ ^18^F]FDG‐PET Study on Sex‐Related Differences

**DOI:** 10.1111/ene.70412

**Published:** 2025-11-07

**Authors:** Antonio Canosa, Stefano Callegaro, Umberto Manera, Rosario Vasta, Sara Cabras, Francesca Di Pede, Filippo De Mattei, Francesca Palumbo, Barbara Iazzolino, Anastasia Dei Giudici, Enrico Matteoni, Grazia Zocco, Emilio Minerva, Alessandra Maccabeo, Giorgio Pellegrino, Daniela Pascariu, Maurizio Grassano, Francesco Ciresi, Marcella Testa, Giulia Polverari, Paolina Salamone, Giovanni De Marco, Claudia Paolantonio, Giulia Marchese, Cristina Moglia, Andrea Calvo, Adriano Chiò, Marco Pagani

**Affiliations:** ^1^ ALS Centre, ‘Rita Levi Montalcini’ Department of Neuroscience University of Turin Turin Italy; ^2^ Azienda Ospedaliero‐Universitaria Città della Salute e della Scienza di Torino Neurology Unit 1U Turin Italy; ^3^ Institute of Cognitive Sciences and Technologies C.N.R. Rome Italy; ^4^ Neuroscience Institute of Turin (NIT) Turin Italy; ^5^ Centre for Neuroscience University of Camerino Camerino Italy; ^6^ Positron Emission Tomography Centre AFFIDEA‐IRMET S.p.A. Turin Italy; ^7^ Department of Medical Radiation Physics and Nuclear Medicine Karolinska University Hospital Stockholm Sweden

**Keywords:** 2‐[^18^F]FDG‐PET, amyotrophic lateral sclerosis, cognition, cognitive reserve, sex

## Abstract

**Background:**

Cognitive reserve (CR) applies to ALS‐related cognitive impairment and education is a CR proxy. The influence of sex on CR in ALS is unclear.

**Methods:**

We compared brain 2‐[^18^F]FDG‐PET metabolism of male (m‐ALS, *n* = 95) and female (f‐ALS, *n* = 95) patients, matched for age, education, onset, and King's stage, with no significant difference in ECAS scores. In each group, clusters showing a negative/positive correlation with education were used as seed regions in an interregional correlation analysis (IRCA) to evaluate connectivity. We identified the seed regions including age, onset, King's stage and ECAS as covariates.

**Results:**

M‐ALS showed a relative hypometabolism compared to f‐ALS in bilateral frontotemporal regions. In f‐ALS brain metabolism positively correlated with education in the left fusiform gyrus, cerebellum and pons. The IRCA showed a positive correlation of the seed region with the cerebellum, pons, right fusiform gyrus and cuneus, and the left precuneus, and a negative correlation with the frontal lobes and caudate nuclei. In m‐ALS brain metabolism negatively correlated with education in the left frontotemporal and insular cortices. The IRCA showed a positive correlation of the seed region with bilateral frontotemporal and cingulate cortices, and the right parietal cortex, and a negative correlation with bilateral cerebellum and motor cortex, and the left lingual gyrus.

**Conclusions:**

M‐ALS showed relative frontotemporal hypometabolism compared to f‐ALS, suggesting a male prevalence of CR. In m‐ALS the negative correlation of education with left frontotemporal and insular metabolism supports the CR hypothesis. In f‐ALS the positive correlation of cerebellar metabolism with education suggests compensatory mechanisms, also supported by the IRCA.

## Graphical abstract

We aimed to evaluate sex‐related differences of Cognitive Reserve (CR) in the area of cognitive impairment associated with ALS using brain 2‐[^18^F]FDG‐PET. In male‐ALS the negative correlation of education with left frontotemporal and insular metabolism supports the CR hypothesis. In female‐ALS the positive correlation of cerebellar metabolism with education suggests compensatory mechanisms. The comprehension of the neurobiology underpinning CR could lead to prevention and treatment strategies based on environmental enrichment (EE), and to the development of drugs mimicking the effect of EE (i.e., enviromimetics).

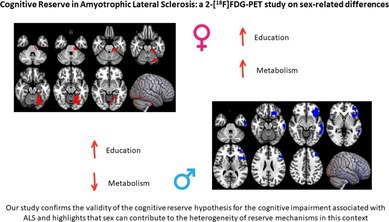

## 1 Introduction

Amyotrophic lateral sclerosis (ALS) is a neurodegenerative disorder affecting both sexes that involves upper and lower motor neurons at the bulbar and spinal level. Frontotemporal dementia (FTD) is observed in about 15% of patients with ALS, with milder cognitive and behavioral deficits occurring in approximately 35% of cases [1]. The determinants of cognitive impairment associated with ALS include genetic factors, while the influence of lifestyle is a subject of debate. Among ALS‐related genes, *C9ORF72* is the most frequent cause of genetic ALS and is strongly associated with cognitive and neuropsychiatric symptoms [2]. As regards modifiable factors, education seems to contribute to cognitive reserve (CR) in ALS patients [3]. First, reserve mechanisms have been hypothesised to explain the discrepancy between neuropathological findings and clinical phenotype in patients with dementia [4]. Over the past decades, the concept of CR has evolved into an active and dynamic model that highlights the flexibility of cognitive processes in the adult brain. This flexibility allows individuals to adapt to brain damage and attenuate or delay the manifestation of symptoms [5]. In a previous study [3] we showed the applicability of the concept of CR to cognitive impairment associated with ALS, particularly the role of education as a CR proxy, but the possible influence of sex on reserve mechanisms was not investigated. Sex‐related differences in CR mechanisms have been explored in healthy aging, Alzheimer's Disease (AD) and FTD. A study investigated sex‐related differences in the effect of education and occupation, as proxies of CR, on brain metabolism assessed by 2‐[^18^F]fluoro‐2‐deoxy‐D‐glucose‐Positron‐Emission Tomography (2‐[^18^F]FDG‐PET) in healthy aging and AD. Among healthy elderly subjects, males showed a greater age‐related decline in brain metabolism than females in frontal medial regions. In both healthy subjects and AD, the effect of education and occupation was stronger in anterior executive control and socio‐affective networks in females, and in posterior associative brain regions and the default mode network in males [6]. A Magnetic Resonance Imaging (MRI) study focused on behavioral FTD showed that women have more severe frontotemporal atrophy than men, despite similar levels of cognitive impairment at diagnosis. On the other hand, women had better‐than‐expected executive function scores and milder changes in apathy, sleep and appetite than men with similar levels of cortical atrophy. These results suggest that women are better able to cope with neurodegenerative changes thanks to a more efficient behavioral and executive reserve than men, and that similar severity of symptoms must be underpinned by a higher neurodegenerative brain burden in women than in men [7].

In this context, we aimed to evaluate sex‐related differences of CR in the area of cognitive impairment associated with ALS using brain 2‐[^18^F]FDG‐PET.

## 2 Material and Methods

### 2.1 Participants

We randomly collected two groups, one composed of male (m‐ALS) and one of female subjects (f‐ALS), from the series of 891 ALS patients who underwent brain 2‐[^18^F]FDG‐PET at diagnosis between 2008 and 2022 at the ALS Centre of Turin, Italy. All patients were diagnosed with definite, probable, and probable laboratory‐supported ALS according to El Escorial revised diagnostic criteria [8]. We used a Python v3.10 script (see S1—Methods) to collect two samples matched for the following characteristics: age at PET (below/above median value of the sample), education (< or ≥ 13 years, corresponding to the completion of high school), site of onset (bulbar/spinal/FTD), King's stage (calculated from ALSFRS‐R) [9]. Subsequently, we used the Mann–Whitney test to confirm the absence of differences in the mentioned variables, in addition to ECAS total score, between the two groups. We matched the two samples for the highest number of variables allowing the collection of adequate‐sized samples. Patients carrying genetic mutations in ALS‐related genes were excluded: although reserve mechanisms seem to be involved even in the presence of an unfavorable genetic status [10], this condition deserves an ad hoc study.

### 2.2 Evaluation of Cognitive Functions

We used the validated Italian version [11] of the Edinburgh Cognitive and Behavioral Amyotrophic Lateral Sclerosis Screen (ECAS) [12]. ECAS was specifically designed as a tool for cognitive screening of ALS patients, including proper corrections for limb and bulbar impairment. It encompasses several cognitive domains, grouped as ALS‐specific (Language, Verbal Fluency, Executive Functions) and non‐ALS specific (Verbal Memory and Visuospatial Abilities), and a brief caregiver interview about behavioral changes, based on current diagnostic criteria for frontotemporal dysfunction in ALS [13]. From the ECAS we obtain two subscores (ALS specific and non‐ALS specific) and a total score (ALS total), indicating the severity of the impairment. The total score was considered a measure of the overall level of cognitive performance in this study. The whole study sample also underwent a full neuropsychological assessment, whose methodological details are described elsewhere [14], to obtain the classification based on the diagnostic criteria for frontotemporal dysfunction in ALS [13].

### 2.3 Cognitive Reserve Proxy

We considered education as a proxy for CR, as consistently reported in the available literature on CR in AD [ 15] and FTD [16] and fruitfully done in our previous study on CR in ALS [3]. It was calculated as the number of completed years of schooling, adding eventual years of apprenticeship only when formal education was planned. Information provided by patients was verified by their caregivers.

### 2.4 2‐[ ^18^F]FDG‐PET Image Acquisition and Pre‐Processing

Brain 2‐[^18^F]FDG‐PET was performed according to published guidelines [17]. Patients fasted at least 6 h before the exam. Blood glucose was < 7.2 mmol/L in all cases before the procedure. After a 20‐min rest, 185–200 MBq of 2‐[^18^F]FDG was injected. The acquisition started 60 min after the injection. PET/CT scans were performed on a Discovery ST‐E System (General Electric). Brain CT and PET scans were sequentially acquired, the former being used for attenuation correction of PET data. The PET images were reconstructed with four iterations and 28 subsets with an initial voxel size of 2.34 × 2.34 × 2.00 mm, and data were collected in 128 × 128 matrices. Images were spatially normalised to a customised brain 2‐[^18^F]FDG‐PET template [18] and subsequently smoothed with a 10‐mm filter in MATLAB R2018b (MathWorks, Natick, MA, USA). Intensity normalisation was performed at the individual level averaging each voxel for the mean value of the whole brain.

### 2.5 Statistical Analysis

First, we compared the two study groups (i.e., m‐ALS and f‐ALS) through the two‐sample t‐test model of SPM12, setting the height threshold at *p* < 0.001 uncorrected (*p* < 0.05 FWE‐corrected at cluster level). Subsequently, in each study sample separately (i.e., m‐ALS and f‐ALS), education (years) was regressed out against whole‐brain metabolism. The SPM12 Multiple Regression routine was implemented with age at PET, site of onset (spinal/bulbar), King's stage at PET, and ECAS total score at PET as covariates. The height threshold was set at *p* < 0.005 uncorrected (*p* < 0.05 FWE‐corrected at cluster level). In each group separately, metabolic clusters showing a significant negative or positive correlation with education were used as seed regions in a multiple regression analysis to identify cerebral regions whose metabolism was positively or negatively correlated with that of the seed clusters (i.e., interregional correlation analysis, IRCA). The IRCA is a method to evaluate brain metabolic connectivity. In the IRCA, the height threshold was set at *p* < 0.001 uncorrected (*p* < 0.05 FWE‐corrected at cluster level). In all the analyses only clusters containing > 125 contiguous voxels were considered significant. Brodmann areas (BAs) were identified at a 0–2‐mm range from the Talairach coordinates of the SPM output isocenters corrected by Talairach Client (http://www.talairach.org/index.html).

### 2.6 Protocol Approvals

The study was approved by the ethical committee ‘Comitato Etico Interaziendale Azienda Ospedaliero‐Universitaria Città della Salute e della Scienza di Torino’ (Protocol number 0011669). Patients signed a written informed consent. They did not receive any remuneration for participation. The study conforms with the World Medical Association Declaration of Helsinki.

## 3 Results

The study groups (f‐ALS and m‐ALS) were composed of 95 subjects. The demographic and clinical characteristics are reported in Supporting Information (Table S1). We found no difference between f‐ALS and m‐ALS in terms of age at PET, education, site of onset, King's stage, ECAS total score and cognitive categories according to international criteria [13].

### 3.1 2‐[ ^18^F]FDG‐PET Data: Comparison Between f‐ALS and m‐ALS

In the direct comparison between f‐ALS and m‐ALS we identified a relative hypometabolism in m‐ALS in clusters including extensive bilateral frontotemporal regions, as well as more limited right parieto‐occipital regions (Table S2, Figure S1).

### 3.2 2‐[ ^18^F]FDG‐PET Data: F‐ALS

**FIGURE 1:**
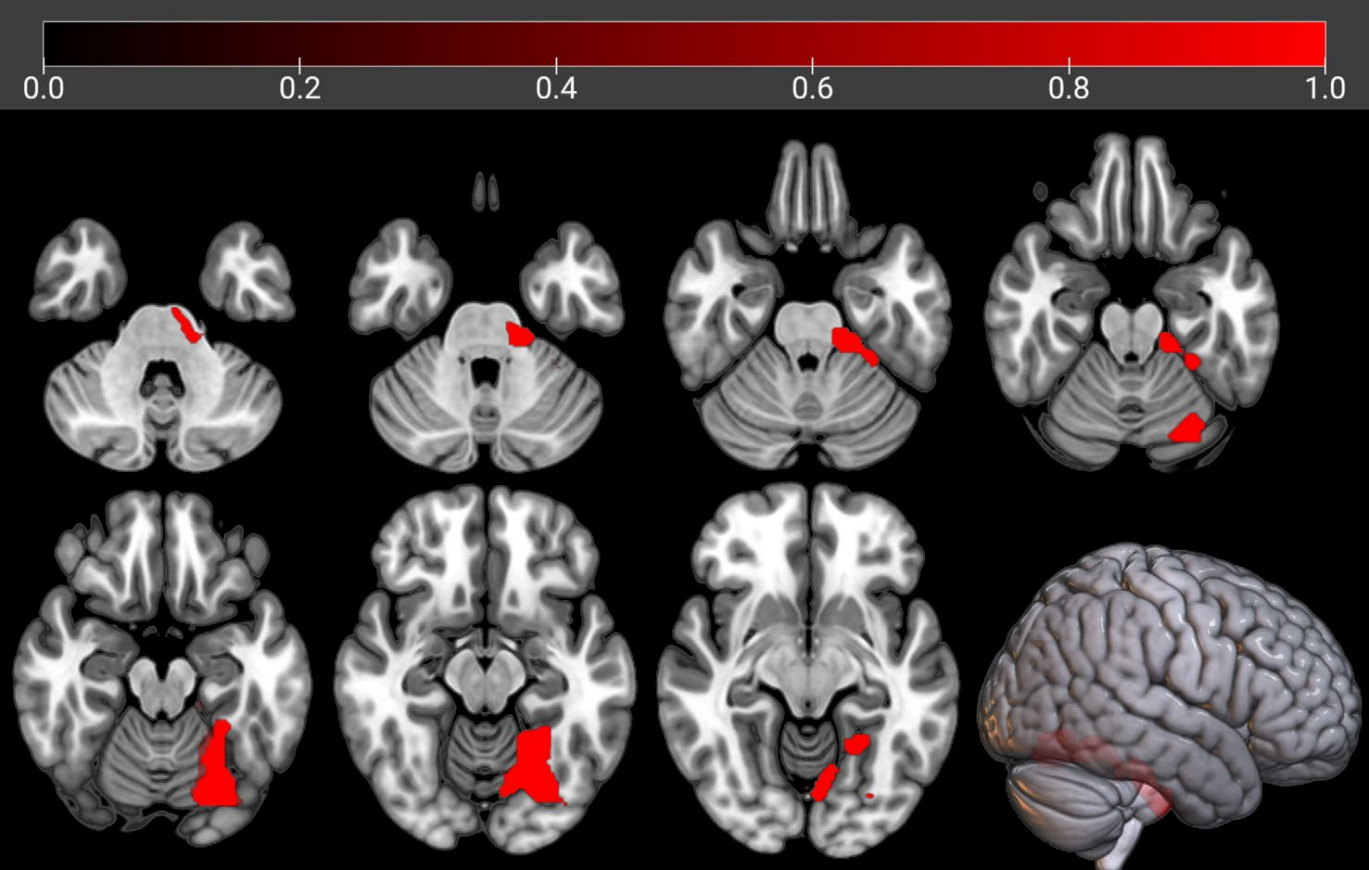
The regions showing a statistically significant positive correlation between brain metabolism and education in f‐ALS are marked in red. The clusters are reported on axial sections of a brain Magnetic Resonance Imaging template and on the brain surface of a glass brain rendering (bottom right).

In f‐ALS we did not find any cluster of negative correlation between brain metabolism and education. Otherwise, we found a positive correlation in a cluster including the left fusiform gyrus, left cerebellar regions, and left pons and middle cerebellar peduncle (Table S3, Figure 1).

**FIGURE 2:**
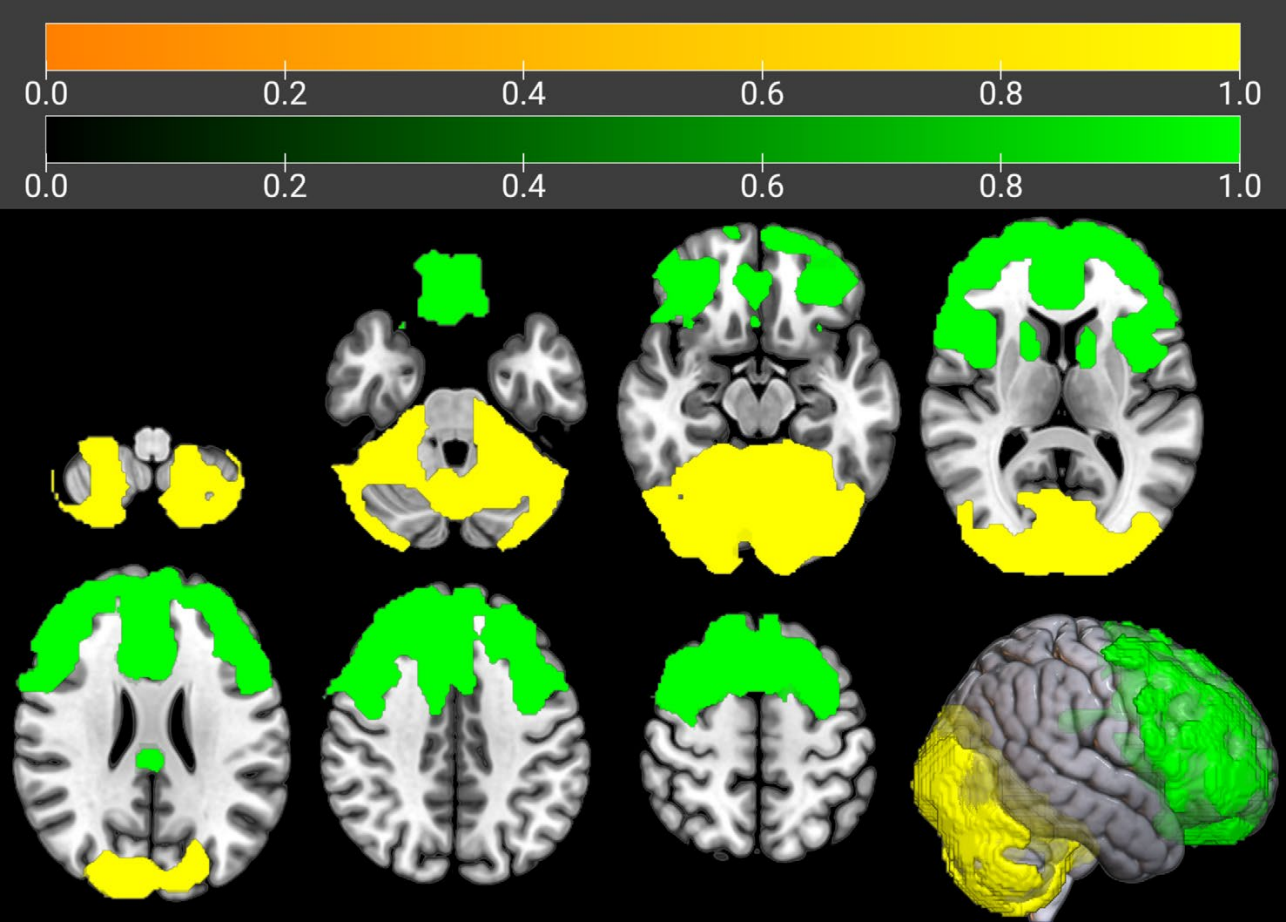
IRCA in f‐ALS: Clusters showing a statistically significant positive (marked in yellow) and negative (marked in green) correlation with the seed region are reported on axial sections of a brain Magnetic Resonance Imaging template and on the brain surface of a glass brain rendering (bottom right).

We used this cluster as the seed region for the IRCA in f‐ALS. In the IRCA we found a positive correlation of seed region metabolism with the bilateral cerebellum, pons, right fusiform gyrus and cuneus, and left precuneus. We found a negative correlation of seed region metabolism with a very large cluster encompassing both frontal lobes and with the bilateral caudate nuclei (Figure 2, Table 1).

**TABLE 1:** IRCA in f‐ALS: Clusters of positive and negative correlations with the seed region.

p FWE – corrected	Cluster extent	*Z* score	Talairach coordinates (*x*, *y*, *z*)			Brain region		
**IRCA in f‐ALS: cluster of positive correlation with the seed region**								
0.000	33,850	65,535	−28.0	−65.0	−12.0	Left cerebellum	Posterior lobe	Declive
		65,535	−24.0	−61.0	−12.0	Left cerebellum	Posterior lobe	Declive
		7.55	−30.0	−44.0	−20.0	Left cerebellum	Anterior lobe	Culmen
		7.28	22.0	−53.0	−11.0	Right cerebellum	Posterior lobe	Declive
		7.14	18.0	−65.0	−9.0	Right cerebellum	Posterior lobe	Declive
		6.86	38.0	−78.0	−13.0	Right cerebrum	Occipital lobe	Fusiform gyrus, BA 19
		6.70	18.0	−84.0	−43.0	Right cerebellum	Posterior lobe	Inferior semi‐lunar lobule
		6.38	6.0	−88.0	23.0	Right cerebrum	Occipital lobe	Cuneus, BA 18
		5.96	8.0	−101.0	0.0	Right cerebrum	Occipital lobe	Cuneus, BA 17
		5.88	−24.0	−76.0	−48.0	No gray matter found		
		5.79	30.0	−75.0	−50.0	No gray matter found		
		5.79	−32.0	−80.0	−45.0	Left cerebellum	Posterior lobe	Inferior semi‐lunar lobule
		5.72	28.0	−78.0	−48.0	No gray matter found		
		5.59	−36.0	−78.0	−45.0	Left cerebellum	Posterior lobe	Inferior semi‐lunar lobule
		5.59	30.0	−37.0	−39.0	Right cerebellum	Posterior lobe	Cerebellar tonsil
		5.54	−22.0	−78.0	24.0	Left cerebrum	Occipital lobe	Precuneus, BA 31
**IRCA in f‐ALS: cluster of negative correlation with the seed region**								
0.000	41,426	6.78	−28.0	7.0	57.0	Left cerebrum	Frontal lobe	Middle frontal gyrus, BA 6
		6.75	38.0	5.0	57.0	Right cerebrum	Frontal lobe	Middle frontal gyrus, BA 6
		6.70	26.0	8.0	53.0	Right cerebrum	Frontal lobe	Superior frontal gyrus, BA 6
		6.47	38.0	16.0	45.0	Right cerebrum	Frontal lobe	Middle frontal gyrus, BA 8
		6.29	18.0	32.0	50.0	Right cerebrum	Frontal lobe	Superior frontal gyrus, BA 8
		6.22	−32.0	56.0	−1.0	Left cerebrum	Frontal lobe	Superior frontal gyrus, BA 10
		6.18	46.0	15.0	34.0	Right cerebrum	Frontal lobe	Middle frontal gyrus, BA 9
		6.08	46.0	19.0	27.0	Right cerebrum	Frontal lobe	Middle frontal gyrus, BA 46
		5.91	18.0	58.0	27.0	Right cerebrum	Frontal lobe	Superior frontal gyrus, BA 9
		5.80	8.0	40.0	29.0	Right cerebrum	Frontal lobe	Medial frontal gyrus, BA 9
		5.65	−6.0	52.0	38.0	Left cerebrum	Frontal lobe	Medial frontal gyrus, BA 9
		5.65	50.0	15.0	20.0	Right cerebrum	Frontal lobe	Inferior frontal gyrus, BA 9
		5.64	−6.0	62.0	2.0	Left cerebrum	Frontal lobe	Medial frontal gyrus, BA 10
0.040	431	4.32	16.0	14.0	3.0	Right cerebrum	Sub‐lobar	Caudate head
0.032	463	4.05	−14.0	4.0	11.0	Left cerebrum	Sub‐lobar	Caudate body
		4.01	−10.0	14.0	−1.0	Left cerebrum	Sub‐lobar	Caudate head

Abbreviation: BA, Brodmann area.

### 3.3 2‐[ ^18^F]FDG‐PET Data: M‐ALS

**FIGURE 3:**
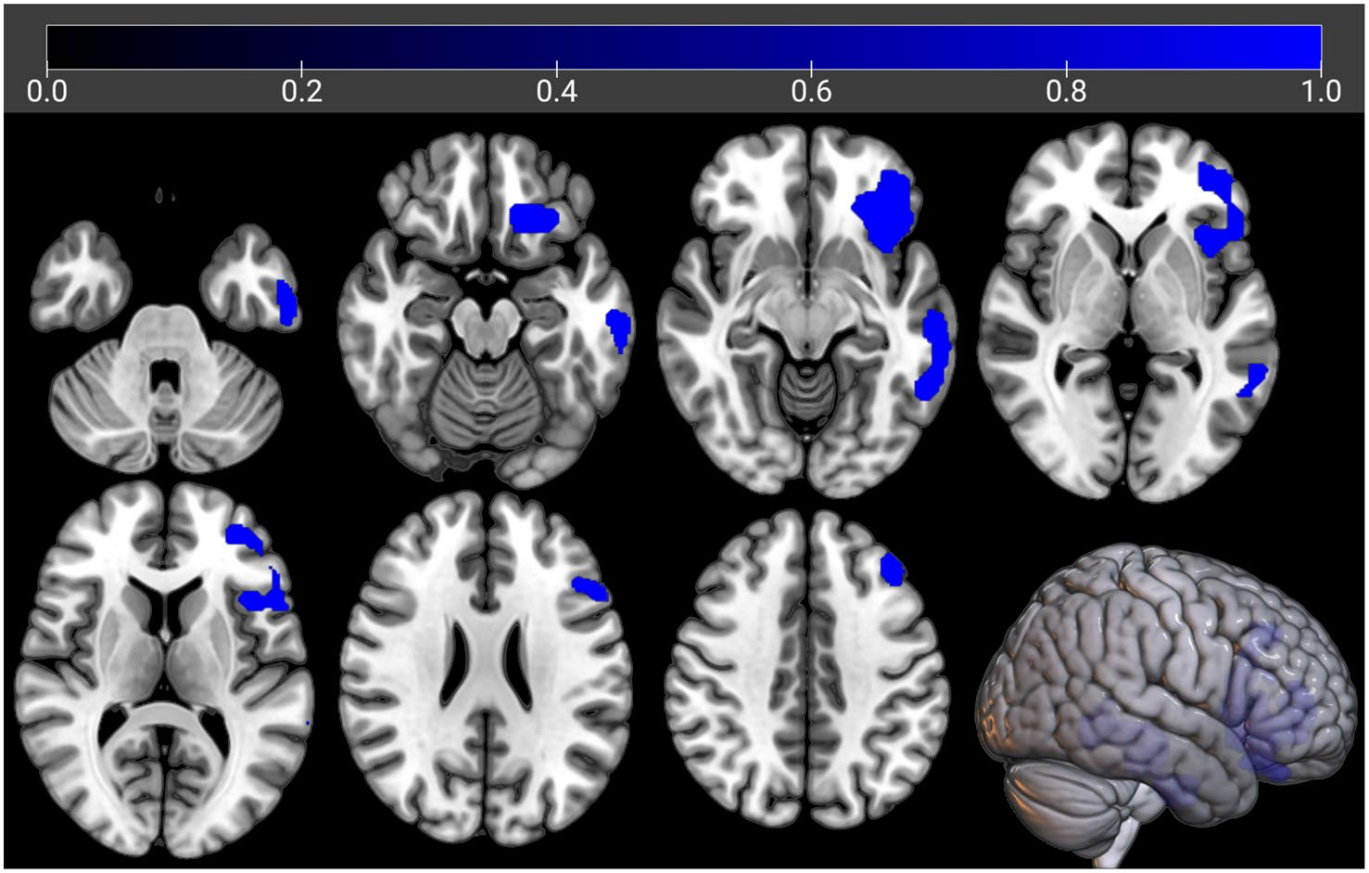
The regions showing a statistically significant negative correlation between brain metabolism and education in m‐ALS are marked in blue. The clusters are reported on axial sections of a brain Magnetic Resonance Imaging template and on the brain surface of a glass brain rendering (bottom right).

In m‐ALS we did not find any cluster of positive correlation between brain metabolism and education. Otherwise, we found a negative correlation between brain metabolism and education in clusters including left frontotemporal and insular cortices (Table S4, Figure 3).

**FIGURE 4:**
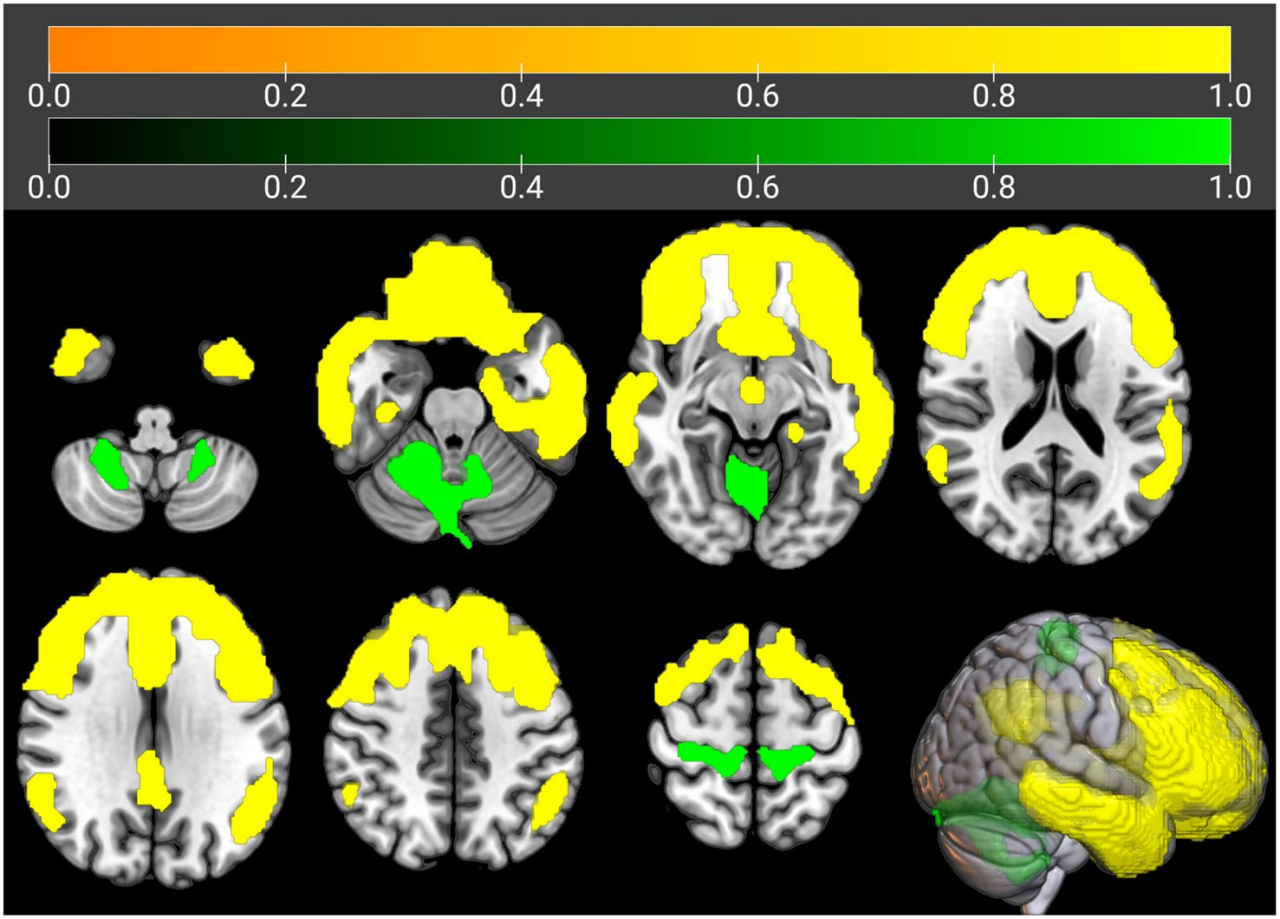
IRCA in m‐ALS: Clusters showing a statistically significant positive (marked in yellow) and negative (marked in green) correlation between brain metabolism and the seed region are reported on axial sections of a brain Magnetic Resonance Imaging template and on the brain surface of a glass brain rendering (bottom right).

We used this cluster as the seed region for the IRCA in m‐ALS. In the IRCA we found a positive correlation of seed region metabolism with bilateral frontotemporal cortices, right parietal cortex, and bilateral cingulate cortex. We found a negative correlation of seed region metabolism with bilateral cerebellar clusters, left lingual gyrus, and bilateral motor cortex (Figure 4, Table 2).

**TABLE 2:** IRCA in m‐ALS: Clusters of positive and negative correlation with the seed region.

p FWE – corrected	Cluster extent	*Z* score	Talairach coordinates (x, y, z)			Brain region		
**IRCA in m‐ALS: cluster of positive correlation with the seed region**								
0.000	66,166	65,535	−38.0	23.0	−10.0	Left cerebrum	Frontal lobe	Inferior frontal gyrus, BA 47
		65,535	−42.0	46.0	−9.0	Left cerebrum	Frontal lobe	Middle frontal gyrus, BA 11
		65,535	−46.0	39.0	−4.0	Left cerebrum	Frontal lobe	Middle frontal gyrus, BA 47
		65,535	−50.0	24.0	17.0	Left cerebrum	Frontal lobe	Inferior frontal gyrus, BA 45
		65,535	8.0	34.0	−22.0	Right cerebrum	Frontal lobe	Rectal gyrus, BA 11
		65,535	−6.0	40.0	−22.0	Left cerebrum	Frontal lobe	Orbital gyrus, BA 11
		65,535	−20.0	60.0	−10.0	Left cerebrum	Frontal lobe	Superior frontal gyrus, BA 11
		65,535	−10.0	59.0	−15.0	Left cerebrum	Frontal lobe	Medial frontal gyrus, BA 11
		65,535	48.0	40.0	−10.0	Right cerebrum	Frontal lobe	Inferior frontal gyrus, BA 47
		65,535	−42.0	38.0	15.0	Left cerebrum	Frontal lobe	Middle frontal gyrus, BA 46
		65,535	−63.0	−26.0	−9.0	Left cerebrum	Temporal lobe	Middle temporal gyrus, BA 21
		65,535	−44.0	25.0	30.0	Left cerebrum	Frontal lobe	Middle frontal gyrus, BA 9
0.001	1067	4.66	57.0	−49.0	26.0	Right cerebrum	Temporal lobe	Supramarginal gyrus, BA 40
		4.59	55.0	−43.0	32.0	Right cerebrum	Parietal lobe	Supramarginal gyrus, BA 40
		3.89	50.0	−60.0	34.0	Right cerebrum	Parietal lobe	Angular gyrus, BA 39
0.004	790	4.21	4.0	−29.0	33.0	Right cerebrum	Limbic lobe	Cingulate gyrus, BA 31
		3.98	−4.0	−49.0	28.0	Left cerebrum	Limbic lobe	Cingulate gyrus, BA 31
**IRCA in m‐ALS: cluster of negative correlation with the seed region**								
0.000	4042	5.25	10.0	−63.0	−10.0	Right cerebellum	Anterior lobe	Culmen
		4.91	−2.0	−84.0	−13.0	Left cerebrum	Occipital lobe	Lingual gyrus, BA 18
		4.18	12.0	−66.0	−32.0	Right cerebellum	Posterior lobe	Uvula
		4.09	24.0	−49.0	−38.0	Right cerebellum	Posterior lobe	Cerebellar tonsil
		3.90	−26.0	−45.0	−36.0	Left cerebellum	Posterior lobe	Cerebellar tonsil
		3.26	−12.0	−66.0	−32.0	Left cerebellum	Posterior lobe	Uvula
0.000	1787	4.70	10.0	−28.0	66.0	Right cerebrum	Frontal lobe	Medial frontal gyrus, BA 6
		4.54	−4.0	−24.0	66.0	Left cerebrum	Frontal lobe	Medial frontal gyrus, BA 6
		4.46	−14.0	−28.0	60.0	Left cerebrum	Frontal lobe	Precentral gyrus, BA 4
		4.02	26.0	−26.0	58.0	Right cerebrum	Frontal lobe	Precentral gyrus, BA 4

Abbreviation: BA, Brodmann area.

## 4 Discussion

Our study provides novel evidence regarding sex‐specific patterns of CR in ALS patients, expanding previous findings on CR mechanisms in this disease. Our data reveal that male patients show higher metabolic impairment (i.e., a relative hypometabolism) than female subjects with overlapping cognitive status, mainly in frontotemporal regions, suggesting a male prevalence of reserve mechanisms. Based on these findings, m‐ALS seem to cope better with a more severe cerebral lesion load in frontotemporal cortices, which are typically affected in cognitive impairment associated with ALS [19]. This hypothesis is in agreement with population‐based data pointing out that women with ALS are more affected by cognitive impairment than men at increasing ages [2]. Reserve mechanisms may be based on a greater functional reserve in younger subjects than in older subjects [5]. Nevertheless, in the present study we included two groups of comparable age and the effect of this variable on the results was excluded by including it as a covariate. Therefore, our results appear to be sex‐specific. However, further studies are warranted to evaluate the role of age in modulating CR.

In order to assess eventual differences in the substratum of CR in m‐ALS and f‐ALS, we evaluated the relationship between brain metabolism and education as CR proxy in each group separately. The results obtained in the two groups represent two sides of the same coin. In m‐ALS we identified a negative correlation of years of schooling with left frontotemporal and insular clusters, which are typically affected in ALS‐related cognitive impairment [19], in line with the results of seminal studies supporting the CR hypothesis in AD [20, 21]. Indeed this inverse relationship between brain metabolism and the CR proxy strengthens the idea that highly educated subjects show a greater resilience to the neurodegenerative process. On the other hand, in f‐ALS we found a positive correlation between education and brain metabolism in a cluster encompassing left cerebellum, pons and fusiform gyrus. The positive correlation between CR proxies and brain metabolism is considered important to elucidate their relative contribution to increased CR [22]. A relative increase of cerebellar and brainstem metabolism has been reported in association with ALS‐related cognitive impairment [23, 24] and its increase with higher education attainment could be the basis of a compensatory mechanism.

Through the IRCA we evaluated the metabolic connectivity of the clusters showing negative or positive correlation with education in f‐ALS and m‐ALS respectively, in order to further elucidate brain networks underlying CR. In the IRCA in f‐ALS, the seed region (i.e., left fusiform gyrus, left cerebellar regions, and left pons and middle cerebellar peduncle) showed a negative correlation with extensive bilateral frontal cortices and bilateral caudate. This finding provides further support to the hypothesis of the involvement of the cerebellum in compensatory strategies, since the frontal cortex and caudate nuclei show particular vulnerability in ALS patients with cognitive impairment [19, 25]. On the other hand, in f‐ALS the seed region showed a positive correlation with the bilateral cerebellum, pons, right fusiform gyrus and cuneus, and left precuneus, far beyond the expected autocorrelation, especially at the cerebellar level. While we have hypothesized a compensatory role for the cerebellum, the interpretation of the metabolic connectivity between the cerebellum and the cuneus/precuneus is more challenging and conclusions cannot be drawn. Cuneus and precuneus are part of the Default Mode Network (DMN), which is made up of the regions of the brain that are active when a person is awake and alert, but not actively engaged in an attentional or goal‐directed task [26]. Connectivity changes between the cerebellum and the DMN have been reported in other clinical conditions, including schizophrenia, depression and post‐traumatic stress disorder [27]. Nevertheless, the involvement of the DMN in the ALS‐FTD continuum is not fully understood. In particular, resting‐state functional MRI studies comparing DMN changes in ALS and FTD have shown divergent connectivity patterns in the two syndromes at early stages, namely reduced connectivity in its posterior part in ALS and in its frontal part in FTD, interpreted as reflecting different pathophysiological courses along the ALS‐FTD spectrum [28].

The IRCA in m‐ALS revealed a positive correlation of the frontotemporal seed region metabolism with that of large bilateral frontotemporal cortices. This finding is in line with the fact that these regions share hypometabolic changes in frontotemporal cognitive decline [29]. As regards the negative correlation of the seed region with cerebellar metabolism, the hypothesis of a cerebellar compensatory role has been already discussed. Conversely, the inverse correlation between frontotemporal and motor cortex metabolism is challenging to explain. In the context of ALS, a similar finding has been reported in presymptomatic individuals carrying the *C9ORF72* expansion, who showed the coexistence of prefrontal hypometabolism and motor cortex hypermetabolism [30]. The interpretation of the increased glucose metabolism could be disentangled by combining 2‐[^18^F]FDG with tracers targeting neuroinflammation. Indeed, studies in animal models of other neurodegenerative diseases, such as synucleinopathies, have shown that neurodegenerative changes can lead to alternating patterns of hyper‐ and hypometabolism in the brain, with increased 2‐[^18^F]FDG uptake corresponding to an inflammatory response [31].

Taken together, our results suggest that CR can play a role in both female and male ALS subjects, probably with different mechanisms, in agreement with previous studies on healthy elderly and AD patients [6]. This heterogeneity in CR mechanisms could be underpinned by adaptive changes at the cellular and molecular level in addition to network reorganization [32]. The future combination of 2‐[^18^F]FDG with tracers evaluating the synaptic density [33] and the circuitry associated with specific neurotransmitters [34] might shed light on this unsolved issue. As for the possible compensatory role of the cerebellum, the data from the present study do not definitely clarify whether it is part of a functional network physiologically present in healthy subjects (i.e., the brain reserve component of CR) or it is recruited in the presence of the neurodegenerative process due to ALS (i.e., the brain compensation component of CR). Nevertheless, our previous study on CR including both ALS patients and healthy subjects suggests that the cerebellum comes into play in the presence of ALS‐related brain damage [3].

Our study has some limitations. First, we did not consider some well‐established proxies of CR, such as occupation, leisure activities, and social engagement [35]. Nevertheless, education has been consistently demonstrated to be an independent proxy of CR in several disorders [5]. Second, our measurement of cognitive performance was based on the ECAS, instead of the full neuropsychological assessment. In this respect, in addition to the already mentioned advantages, we should underline the following strengths of the ECAS: it is suitable for use in the clinical setting, as it is not time consuming (the assessment takes approximately 15–20 min); it can also be administered by physicians and nurse specialists, in addition to neuropsychologists [12, 36]; it has been validated in several languages [37]. Third, MRI scans were not available for all subjects. Therefore, we did not perform a partial volume effect (PVE) correction for cortical atrophy. Nevertheless, studies employing voxel‐based atrophy correction of resting glucose metabolism showed that metabolic measurements were relatively independent of brain atrophy [38]. Finally, we acknowledge that the comparison of each patient group with a control group of the same sex would contribute to a better understanding of the issue addressed by our study. Unfortunately, the limited sample size of the control group available at our centre (the same included in our previous study on CR [3]) did not allow this further analysis.

In conclusion, our study confirms the validity of the CR hypothesis for the cognitive impairment associated with ALS and highlights that sex can contribute to the heterogeneity of reserve mechanisms in this context. We warrant further studies to elucidate the interplay between sex and other demographic, genetic and social factors, including age, presence of genetic mutations, occupation and leisure activities, in determining CR in ALS patients. Looking to the long term, the comprehension of the neurobiology underpinning CR could lead to prevention and treatment strategies based on environmental enrichment (EE), and to the development of drugs mimicking the effect of EE (i.e., enviromimetics). In the view of patient‐tailored care, the design of these approaches should consider sex‐related differences [39].

## Author Contributions

*Study concept and design*: Antonio Canosa, Stefano Callegaro, Adriano Chiò, Marco Pagani. *Major role in the acquisition of data*: Antonio Canosa, Stefano Callegaro, Umberto Manera, Rosario Vasta, Sara Cabras, Francesca Di Pede, Filippo De Mattei, Francesca Palumbo, Barbara Iazzolino, Anastasia Dei Giudici, Enrico Matteoni, Grazia Zocco, Emilio Minerva, Alessandra Maccabeo, Giorgio Pellegrino, Daniela Pascariu, Maurizio Grassano, Francesco Ciresi, Marcella Testa, Giulia Polverari, Paolina Salamone, Giovanni De Marco, Claudia Paolantonio, Giulia Marchese, Cristina Moglia, Andrea Calvo. *Analysis and interpretation of data*: Antonio Canosa, Stefano Callegaro, Adriano Chiò, Marco Pagani. *Drafting of the manuscript*: Antonio Canosa, Adriano Chiò, Marco Pagani. *Revision of the manuscript for important intellectual content*: Stefano Callegaro, Umberto Manera, Rosario Vasta, Sara Cabras, Francesca Di Pede, Filippo De Mattei, Francesca Palumbo, Barbara Iazzolino, Anastasia Dei Giudici, Enrico Matteoni, Grazia Zocco, Emilio Minerva, Alessandra Maccabeo, Giorgio Pellegrino, Daniela Pascariu, Maurizio Grassano, Francesco Ciresi, Marcella Testa, Giulia Polverari, Paolina Salamone, Giovanni De Marco, Claudia Paolantonio, Giulia Marchese, Cristina Moglia, Andrea Calvo. *Study supervision*: Adriano Chiò, Marco Pagani. *Obtained funding*: Adriano Chiò, Marco Pagani.

## Conflicts of Interest

Andrea Calvo has received a research grant from Cytokinetics. Adriano Chiò serves on scientific advisory boards for Mitsubishi Tanabe, Roche, Biogen, Cytokinetics, Denali Therapeutics, Amylyx, Eli‐Lilly, and AveXis. The other authors report no competing interests.

## Supporting information


**Appendix S1:** ene70412‐sup‐0001‐supinfo.docx.

## Data Availability

The script used for patient matching is reported in the Supporting Information (Methods) and the NIfTI files of the clusters identified in the analyses are available on demand by interested researchers.
