# Supplementary material for: Cognitive Reserve in Amyotrophic Lateral Sclerosis: A 2‐[ 18F]FDG‐PET Study on Sex‐Related Differences
Source: Eur J Neurol. 2025 Nov 7;32(11):e70412. doi: 10.1111/ene.70412 (PMC12593548; doi:10.1111/ene.70412)

**Supporting Information - Methods**

We report the script used for patient matching.

import pandas as pd

import numpy as np

from scipy import stats

# Read the Excel file

file_path = DATABASE_FILE_PATH

df = pd.read_excel(file_path)

#remove missing ECAS

df = df[df['TOT ECAS'].notna()]

df = df[df['other genes'] == 'WT']

df = df[df['KINGS TOT'].notna()]

df = df[df['C9ORF=1'] != 1]

df['age-pet-round'] = np.round(df['age-pet-round'],0)

#refactor conditions

cond = [

df['Edu'] < 13,

df['Edu'] >= 13

]

val = [1,2]

df['RANGE_EDU'] = np.select(cond, val)

cond = [

df['age-pet-round'] < 65,

df['age-pet-round'] >= 65

]

val = [1,2]

df['RANGE_AGE'] = np.select(cond, val)

df['KINGS TOT'] = df['KINGS TOT'].astype(int)

cond = [

df['KINGS TOT'] < 2,

df['KINGS TOT'] >= 2

]

val = [1,2]

df['RANGE_KINGS'] = np.select(cond, val)

df['RANGE_ONSET'] = df['ONSET (BUL=0, SPI=1, FTD=2)'].astype(int)

# Define the columns to match

columns_to_match = ['RANGE_EDU', 'RANGE_AGE', 'RANGE_ONSET', 'RANGE_KINGS']

sex_column = 'SEX (M=1, F=2)'

df['match_key'] = df[columns_to_match].astype(str).agg('-'.join, axis=1)

# divide by sex

males = df[df[sex_column] == 1]

females = df[df[sex_column] == 2]

# 1 a 1 matching

matched_pairs = []

for _, male in males.iterrows():

matching_females = females[

(females['match_key'] == male['match_key'])

]

if not matching_females.empty:

# Select the first female matched

female = matching_females.iloc[0]

matched_pairs.append((male, female))

# Remove the selected female

females = females.drop(female.name)

groups = pd.DataFrame([pair[0] for pair in matched_pairs] + [pair[1] for pair in matched_pairs])

group1 = groups[groups['SEX (M=1, F=2)'] == 1]

group2 = groups[groups['SEX (M=1, F=2)'] == 2]

# Save data in Excel

group1.sort_values(by='PET_HDR').to_excel('males.xlsx', index=False)

group2.sort_values(by='PET_HDR').to_excel('females.xlsx', index=False)

df.to_excel('df.xlsx', index=False)

# Calculate pvalues

stat, p_value = stats.mannwhitneyu(group1['age-pet-round'], group2['age-pet-round'])

stat, p_value = stats.mannwhitneyu(group1[~group1['Edu'].isna()]['Edu'], group2[~group2['Edu'].isna()]['Edu'])

stat, p_value = stats.mannwhitneyu(group1['TOT ECAS'], group2['TOT ECAS'])

**Supporting Information - Table 1.** Demographic and clinical characteristics of patient groups.

|  | **m-ALS**  **(n=95)** | **f-ALS**  **(n=95)** | **p-value** |
| --- | --- | --- | --- |
| Cognitive classification, [n (%)] |  |  |  |
| - ALS-bi | 7 (7) | 7 (7) |  |
| - ALS-ci | 14 (15) | 19 (25) |  |
| - ALS-cbi | 7 (7) | 7 (7) | 0.728 |
| - ALS-CN | 62 (65) | 55 (58) |  |
| - ALS-FTD | 3 (3) | 3 (3) |  |
| - Missing | 2 (3) | 0 (0) |  |
| Age at PET, years [median (IQR)] | 66 (57.5-72) | 66 (56-71.5) | 0.774 |
| Diagnostic delay, years [median (IQR)] | 0.7 (0.5-1.1) | 0.8 (0.5-1.3) | 0.492 |
| Δ ALSFRS-R, points [median (IQR)] | 0.8 (0.5-1-3) | 0.8 (0.4-1.3) | 0.490 |
| Onset site |  |  |  |
| - Bulbar | 28 (29) | 28 (29) | 1.000 |
| - Spinal | 67 (71) | 67 (71) |  |
| Education, years [median (IQR)] | 12 (8-13) | 12 (8-13) | 0.820 |
| King’s stage |  |  |  |
| - 1 | 31 (33) | 31 (33) |  |
| - 2 | 36 (38) | 31 (33) | 0.457 |
| - 3 | 22 (23) | 30 (32) |  |
| - 4 | 6 (6) | 3 (2) |  |
| ECAS, points [median (IQR)] | 102 (86-113) | 107 (90-114.5) | 0.363 |

Data were compared using Mann Whitney U test for continuous variable and chi-squared test for categorical variables.

m-ALS: male ALS. f-ALS: female ALS. ALS-bi: ALS with behavioural impairment. ALS-ci: ALS with cognitive impairment. ALS-cbi: ALS with cognitive and behavioural impairment. ALS-CN: ALS with normal cognitive function. ALS-FTD: ALS with Frontotemporal Dementia. IQR: Interquartile Range.

**Supporting Information - Table 2.** Clusters of relative hypometabolism in m-ALS compared to f-ALS. BA: Brodmann area.

| p FWE - corrected | Cluster extent | Z score | Talairach coordinates (x, y, z) | | | Brain region | | |
| --- | --- | --- | --- | --- | --- | --- | --- | --- |
| 0.000 | 22821 | 6.68 | 67.0 | -7.0 | -22.0 | Right Cerebrum | Temporal Lobe | Inferior Temporal Gyrus, BA 21 |
|  |  | 6.39 | 73.0 | -33.0 | -8.0 | Right Cerebrum | Temporal Lobe | Middle Temporal Gyrus, BA 21 |
|  |  | 6.04 | 50.0 | -45.0 | 2.0 | Right Cerebrum | Temporal Lobe | Middle Temporal Gyrus, BA 22 |
|  |  | 5.67 | -50.0 | -41.0 | 2.0 | Left Cerebrum | Temporal Lobe | Middle Temporal Gyrus, BA 22 |
|  |  | 5.66 | -67.0 | -26.0 | -17.0 | Left Cerebrum | Temporal Lobe | Inferior Temporal Gyrus, BA 20 |
|  |  | 5.55 | 30.0 | -101.0 | 2.0 | Right Cerebrum | Occipital Lobe | Middle Occipital Gyrus, BA 18 |
|  |  | 5.41 | 20.0 | -105.0 | 4.0 | Right Cerebrum | Occipital Lobe | Cuneus, BA 18 |
|  |  | 5.29 | 40.0 | -67.0 | 24.0 | Right Cerebrum | Temporal Lobe | Middle Temporal Gyrus, BA 39 |
|  |  | 5.25 | 67.0 | -41.0 | 33.0 | Right Cerebrum | Parietal Lobe | Inferior Parietal Lobule, BA 40 |
|  |  | 5.18 | 46.0 | -85.0 | 19.0 | Right Cerebrum | Occipital Lobe | Middle Occipital Gyrus, BA 19 |
|  |  | 5.14 | 6.0 | -76.0 | 31.0 | Right Cerebrum | Occipital Lobe | Cuneus, BA 19 |
|  |  | 5.11 | -12.0 | -29.0 | 35.0 | Left Cerebrum | Limbic Lobe | Cingulate Gyrus, BA 31 |
|  |  | 5.11 | 22.0 | -61.0 | 55.0 | Right Cerebrum | Parietal Lobe | Superior Parietal Lobule, BA 7 |
| 0.003 | 923 | 5.46 | 55.0 | 37.0 | -7.0 | Right Cerebrum | Frontal Lobe | Inferior Frontal Gyrus, BA 47 |
|  |  | 5.33 | 63.0 | 22.0 | 14.0 | Right Cerebrum | Frontal Lobe | Inferior Frontal Gyrus, BA 45 |
|  |  | 4.51 | 46.0 | 54.0 | -11.0 | Right Cerebrum | Frontal Lobe | Middle Frontal Gyrus, BA 10 |
| 0.000 | 4931 | 5.23 | 40.0 | 50.0 | 25.0 | Right Cerebrum | Frontal Lobe | Superior Frontal Gyrus, BA 10 |
|  |  | 4.78 | -4.0 | 30.0 | 52.0 | Left Cerebrum | Frontal Lobe | Superior Frontal Gyrus, BA 8 |
|  |  | 4.24 | 12.0 | 55.0 | 14.0 | Right Cerebrum | Frontal Lobe | Medial Frontal Gyrus, BA 10 |
|  |  | 4.04 | 0.0 | 38.0 | 31.0 | Left Cerebrum | Frontal Lobe | Medial Frontal Gyrus, BA 9 |
|  |  | 3.94 | -6.0 | 60.0 | 4.0 | Left Cerebrum | Frontal Lobe | Medial Frontal Gyrus, BA 10 |
|  |  | 3.88 | 4.0 | 19.0 | 29.0 | Right Cerebrum | Limbic Lobe | Cingulate Gyrus, BA 24 |
|  |  | 3.79 | 40.0 | 27.0 | 39.0 | Right Cerebrum | Frontal Lobe | Middle Frontal Gyrus, BA 8 |
|  |  | 3.74 | 10.0 | 32.0 | 50.0 | Right Cerebrum | Frontal Lobe | Superior Frontal Gyrus, BA 6 |
|  |  | 3.62 | 40.0 | 29.0 | 28.0 | Right Cerebrum | Frontal Lobe | Middle Frontal Gyrus, BA 9 |
| 0.022 | 564 | 5.18 | -46.0 | 48.0 | -7.0 | Left Cerebrum | Frontal Lobe | Middle Frontal Gyrus, BA 47 |
|  |  | 3.65 | -38.0 | 35.0 | 6.0 | Left Cerebrum | Frontal Lobe | Inferior Frontal Gyrus, BA 46 |

**Supporting Information - Table 3**. Cluster showing a statistically significant positive correlation between brain metabolism and education in f-ALS.

| p FWE - corrected | Cluster  extent | Z  score | Talairach coordinates  (x, y, z) | | | Brain region | | |
| --- | --- | --- | --- | --- | --- | --- | --- | --- |
| 0.027 | 1218 | 3.75 | -16.0 | -30.0 | -20.0 | Left Cerebellum | Anterior Lobe | Culmen |
|  |  | 3.61 | -24.0 | -53.0 | -9.0 | Left Cerebrum | Occipital Lobe | Fusiform Gyrus, BA 19 |
|  |  | 3.60 | -24.0 | -57.0 | -11.0 | Left Cerebellum | Posterior Lobe | Declive |
|  |  | 3.48 | -26.0 | -73.0 | -12.0 | Left Cerebellum | Posterior Lobe | Declive |
|  |  | 3.30 | -6.0 | -19.0 | -33.0 | No gray  matter found | | |
|  |  | 2.94 | -10.0 | -64.0 | -5.0 | Left Cerebellum | Anterior Lobe | Culmen |
|  |  | 2.81 | -30.0 | -40.0 | -18.0 | Left Cerebrum | Temporal Lobe | Fusiform Gyrus, BA 20 |
|  |  | 2.65 | -34.0 | -44.0 | -25.0 | Left Cerebellum | Anterior Lobe | Culmen |

**Supporting Information - Table 4**. Clusters showing a statistically significant negative correlation between brain metabolism and education in m-ALS.

| p FWE -corrected | Cluster extent | Z score | Talairach coordinates  (x, y, z) | | | Brain region | | |
| --- | --- | --- | --- | --- | --- | --- | --- | --- |
| 0.033 | 1199 | 4.51 | -57.0 | -13.0 | -30.0 | Left Cerebrum | Temporal Lobe | Inferior Temporal Gyrus, BA 20 |
|  |  | 3.65 | -55.0 | -51.0 | -4.0 | Left Cerebrum | Temporal Lobe | Middle Temporal Gyrus, BA 21 |
|  |  | 2.80 | -65.0 | -42.0 | 8.0 | Left Cerebrum | Temporal Lobe | Superior Temporal Gyrus, BA 22 |
| 0.000 | 2789 | 4.28 | -36.0 | 21.0 | -9.0 | Left Cerebrum | Frontal Lobe | Inferior Frontal Gyrus, BA 47 |
|  |  | 3.86 | -40.0 | 33.0 | 35.0 | Left Cerebrum | Frontal Lobe | Middle Frontal Gyrus, BA 9 |
|  |  | 3.86 | -44.0 | 45.0 | -2.0 | Left Cerebrum | Frontal Lobe | Inferior Frontal Gyrus, BA 10 |
|  |  | 3.68 | -42.0 | 14.0 | 5.0 | Left Cerebrum | Sub-lobar | Insula, BA 13 |
|  |  | 3.17 | -53.0 | 20.0 | 19.0 | Left Cerebrum | Frontal Lobe | Inferior Frontal Gyrus, BA 45 |
|  |  | 3.03 | -55.0 | 14.0 | 12.0 | Left Cerebrum | Frontal Lobe | Inferior Frontal Gyrus, BA 44 |
|  |  | 2.89 | -42.0 | 23.0 | 25.0 | Left Cerebrum | Frontal Lobe | Middle Frontal Gyrus, BA 46 |

**Supporting Information - Figure 1.** Clusters of relative hypometabolism in m-ALS compared to f-ALS are reported in sky blue on axial sections of a brain Magnetic Resonance Imaging template and on the brain surface of a glass brain rendering (bottom right).


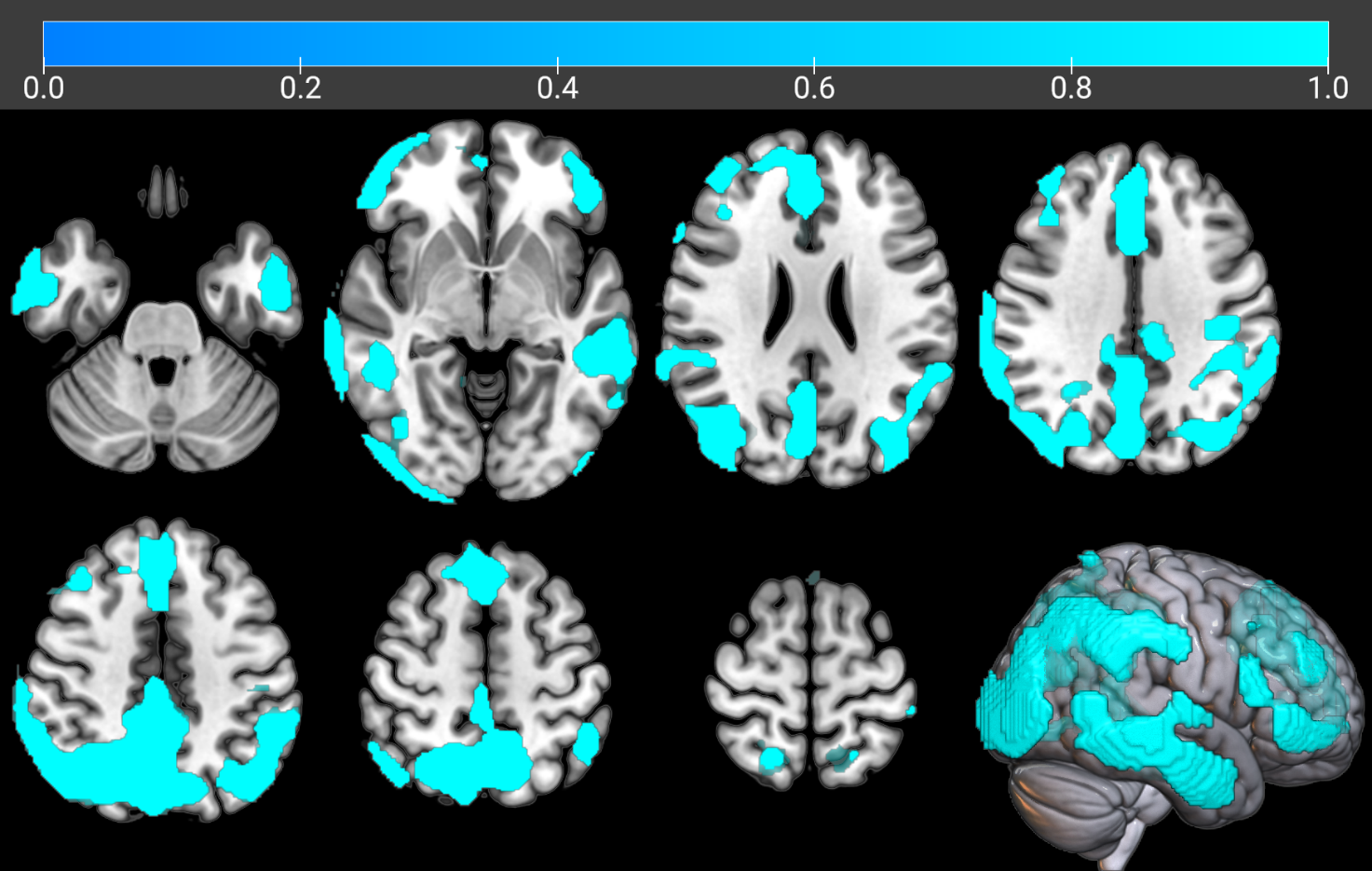

Supplement: Supplementary file 1 — Appendix S1: ene70412‐sup‐0001‐supinfo.docx. [file ENE-32-e70412-s001.docx]
